# Maladaptive sensory coding in the enteric nervous system playing a role in gastrointestinal disorders

**DOI:** 10.3389/fncel.2026.1903390

**Published:** 2026-10-05

**Authors:** Linda Chia-Hui Yu, Ching-Ying Huang, Chia-Ying Lin, Yi-Syuan Tsai, Ying-Chieh Chang, Ching-Wen Huang, Zih-Ling Chao, Wei-Ting Kuo

**Affiliations:** 1 Graduate Institute of Physiology, College of Medicine, National Taiwan University, Taipei, Taiwan; 2 Graduate Institute of Oral Biology, College of Medicine, National Taiwan University, Taipei, Taiwan; 3 Department of Dentistry, National Taiwan University Hospital, Taipei, Taiwan

**Keywords:** dysmotility, enteric nervous system, inflammatory bowel diseases, intrinsic primary afferent neuron, irritable bowel syndrome, neuroimmune communication, secretory reflexes, visceral hypersensitivity

## Abstract

Maladaptive sensory coding within the enteric nervous system (ENS) is increasingly implicated in gastrointestinal dysmotility, abnormal secretion, neuroimmune dysfunction, and visceral hypersensitivity. Intrinsic primary afferent neurons (IPANs), classically corresponding to Dogiel type II/AH neurons, constitute a major sensory arm of intrinsic enteric reflex circuits. They detect mechanical, chemical, epithelial, microbial, and immune-derived signals and relay this information to interneurons, motor neurons, and secretomotor pathways, thereby coordinating gastrointestinal function independently of central input. Experimental studies demonstrate that IPAN excitability is highly plastic: inflammation, serotonergic signaling, cytokines, neurotrophins, microbial products, and other mediators can alter firing thresholds, slow afterhyperpolarization (sAHP), and synaptic transmission. Molecularly defined IPAN populations are sufficient to initiate specific motor programs, microbial colonization is required for normal AH-neuron excitability, and cytokine-responsive subsets participate directly in neuroimmune circuits. These findings support a model in which persistent increases or decreases in sensory gain may distort enteric reflex output and contribute to disorders such as irritable bowel syndrome (IBS), inflammatory bowel disease (IBD), chronic constipation, diarrhea, and visceral pain. However, evidence linking IPAN dysfunction directly to disease remains limited. Few studies have selectively manipulated IPANs in disease models, and definitive demonstrations of IPAN-specific dysfunction causing a gastrointestinal phenotype and its reversal by targeted rescue are lacking, particularly in humans. Major unresolved questions include whether maladaptive IPAN coding is a primary driver, amplifier, or consequence of disease; how intrinsic and extrinsic afferent pathways interact; and how microbial and immune signals reshape IPAN identity over time. Resolving these issues will be essential for establishing maladaptive enteric sensory coding as a therapeutic target.

## Overview of enteric nervous system

The gastrointestinal tract is not merely a digestive and absorptive portal regulated by the brain. The enteric nervous system (ENS) is a highly innervated, self-regulating system with its own sensory-motor machinery. The ENS is embedded within the gut wall, constituting a vast network of neurons and glial cells mostly situated in the myenteric and submucosal plexuses (Goldstein et al., 2013; Furness et al., 2014). This arrangement enables the gut to perceive, analyze, and react to local conditions, without input from the brain. Classic observations demonstrating that isolated intestinal segments can still produce coordinated peristaltic activity after severance of nerve fibers from the brainstem and spinal cord offer compelling evidence that the ENS is not merely a conduit for central commands, but rather an intrinsic neural circuit capable of governing gastrointestinal functions (Spencer and Hu, 2020; Spencer et al., 2016).

The ENS is frequently referred to as the “second brain,” albeit this terminology diminishes its evolutionary and physiological significance. Long before the evolutional emergence of central nervous systems, primitive organisms developed intrinsic neuronal networks that coordinated intake, propulsion, and waste removal (Furness, 2022; Silva et al., 2026; Copenhaver, 2007; Arendt et al., 2015). In mammals, this primordial brain logic has evolved into a complex regulatory system that oversees motility, secretion, absorption, local blood flow, epithelial barrier function, immunological surveillance, and host-microbial communication (Bernardazzi et al., 2016; Schneider et al., 2019; Sharkey and Mawe, 2023; Fung and Vanden Berghe, 2020). The ENS serves as a major integrator of intestinal homeostasis, with crosstalk to mucosal immune cells, enteroendocrine cells, smooth muscle layers, and extrinsic autonomic neurons.

The integrative ability relies on the diversity of intestinal neuronal and glial populations. Enteric motor neurons govern the contraction and relaxation of intestinal smooth muscle (Schneider et al., 2019; Sharkey and Mawe, 2023); interneurons synchronize ascending and descending reflex pathways (Sharkey and Mawe, 2023); enteric glia maintain epithelial integrity, facilitate neuronal signaling, and regulate inflammatory equilibrium (Grubisic and Gulbransen, 2017; Sharkey, 2015; Boesmans et al., 2019). Within these populations, sensory neurons are crucial since they activate local reflex programs in response to mechanical deformation, chemical signals, nutrients, microbial metabolites, inflammatory mediators, and signals generated from epithelial cells (Spencer and Hu, 2020; Cavin et al., 2023; Vicentini et al., 2022; Sun et al., 2025). These sensory components allow the gut to perceive and respond to luminal and tissue information, by generating reflexive actions of peristalsis, secretion, vascular modification, and immune regulation.

The IPAN is a crucial biological component in this sensory apparatus. IPANs can be regarded as the ENS’s local sentinels; they sense alterations in the gut wall and the lumen milieu (Furness et al., 2004; Furness, 2006), subsequently activating enteric circuits independently of brain or spinal cord input (Gershon, 2004). In contrast to extrinsic sensory neurons situated in the dorsal root ganglia or nodose ganglia that convey information from the gut to the central nervous system, IPANs function and transmit signals inside the enteric network itself (Spencer et al., 2026; Hussein et al., 2024). Consequently, they are important for localized, swift, and autonomous reactions in the gastrointestinal tract.

Dysfunction in gastrointestinal sensory neurons can lead to repercussions beyond motility impairment. Defective sensory encoding impairs communication among epithelial cells, immune cells, enteroendocrine cells, and enteric circuits (Sun et al., 2025; Holland et al., 2021). These abnormalities lead to visceral hypersensitivity, aberrant secretion, barrier disruption, inflammation, dysmotility, and modified gut-brain communication (Hussein et al., 2024; Yang et al., 2022; Niesler et al., 2021; Abdullah et al., 2020). Consequently, malfunction of enteric sensory neurons signifies not merely a localized neuronal impairment, but a pathogenic disruption in the gut’s inherent ability to monitor, interpret, and maintain mucosal homeostasis (Niesler et al., 2021).

## Intrinsic primary afferent neuron (IPAN)

IPANs constitute the sensory arm of intrinsic enteric reflex circuits. They detect mechanical, chemical, and thermal stimuli arising within the gut wall and transmit this information to interneurons and motor or secretomotor neurons, thereby initiating local peristaltic, secretory, and vasodilatory reflexes independently of extrinsic innervation. Morphologically, IPANs are classically associated with Dogiel type II neurons, which exhibit smooth, multipolar cell bodies and long circumferentially projecting processes. Electrophysiologically, they largely correspond to AH-type neurons, characterized by a prominent slow after-hyperpolarization that limits repetitive firing and confers a distinctive excitability profile (Brookes et al., 1995). IPAN somata are located predominantly in the myenteric plexus, where they contribute to motility reflexes, but IPAN-like populations are also present in the submucosal plexus. These populations display substantial differences in their neurochemical coding and functional organization (Quinson et al., 2001).

The molecular characterization of IPANs has advanced considerably, although defining markers vary among plexuses, intestinal regions, and species. In the guinea-pig ileum, calbindin immunoreactivity remains one of the most reliable histochemical markers of myenteric Dogiel type II/AH neurons, labeling approximately 82–84% of this population (Quinson et al., 2001). Submucosal neurons were historically considered calbindin-negative; however, more sensitive antisera have revealed calbindin immunoreactivity in approximately 9–10% of these neurons. Notably, these cells largely overlap with cholinergic, calretinin-immunoreactive secretomotor neurons rather than constituting a discrete AH-type sensory population (Quinson et al., 2001). Single-cell and single-nucleus transcriptomic analyses have further refined IPAN classification. Neuromedin U (NMU) has emerged as a prominent conserved marker of putative myenteric IPANs in mice and humans, colocalizing with *Calb2*/*Calcb* in mice and *CALB2*/*SST* in humans (May-Zhang et al., 2021). However, NMU expression is strongly region dependent, identifying approximately 50–75% of putative IPANs in the human small intestine but only 5–10% in the colon, suggesting that additional colonic sensory-neuron subtypes remain to be characterized (May-Zhang et al., 2021). Complementary markers, including *Nefl*, *Klhl1*, *Nxph2*, and *Cdh9*, further distinguish murine myenteric IPAN populations, although their cross-species applicability is limited. For example, *KLHL1* identifies a distinct CALB1-positive type III neuronal population in humans. Moreover, a recently identified submucosal IPAN class exhibits specialized mucosal projections and close anatomical associations with enterochromaffin cells, supporting a potential role in sensing epithelial or luminal signals (Li et al., 2025).

Neurochemically, enteric sensory populations are heterogeneous. Submucosal calbindin-positive neurons are predominantly cholinergic, expressing choline acetyltransferase, and frequently co-express multiple neuropeptides (Quinson et al., 2001). Substance P and other tachykinins, together with calcitonin gene-related peptide (CGRP), commonly colocalize with calbindin, whereas smaller calbindin-positive neurons often express calretinin (Quinson et al., 2001). In the human ileum, putative submucosal IPANs include a population expressing substance P, somatostatin, and CGRP that is distinct from secretomotor neurons expressing vasoactive intestinal peptide (VIP), neuropeptide Y, and calbindin D28k. Myenteric neurons likewise comprise calbindin-positive and NADPH-diaphorase-positive nitrergic populations, with substance P-, somatostatin-, and CGRP-expressing cells forming additional subgroups (Dhatt and Buchan, 1994).

Overall, classical neurochemical studies and contemporary transcriptomic approaches reveal that IPANs represent a heterogeneous sensory-neuron population with marked regional, plexus-specific, and interspecies variation in molecular identity and transmitter repertoire.

## IPANs integrate immune signals to shape enteric function and host defense

IPANs constitute the sensory arm of the ENS, and emerging transcriptomic and functional evidence indicates that their functions extend beyond conventional mechanosensation. IPANs express a broad repertoire of cytokine, chemokine, and pattern-recognition receptors that enables them to directly detect immune activation and inflammatory changes within the intestinal microenvironment. Single-nucleus RNA sequencing and complementary functional studies have identified primary enteric sensory neuron populations expressing receptors for the type 2 cytokines IL-4 and IL-13, with IL-13 signaling mediated through IL-13 receptor α1 (IL-13Rα1) (Wang et al., 2025; Barilla et al., 2025). Beyond type 2 immunity, intrinsic enteric afferents express Toll-like receptors (TLRs), receptors for pro-inflammatory cytokines including TNF, IL-1β, IL-6, and IL-17, and prostaglandin receptors (Benarroch, 2019). Comparatively, vagal capsaicin-sensitive afferents also express receptors for CXCL chemokines and interferons, including IFN-α and IFN-γ, whose cognate ligands can directly activate these sensory fibers (Wang et al., 2017).

This receptor repertoire enables IPANs to translate immune signals derived from macrophages, mast cells, neutrophils, and group 2 innate lymphoid cells (ILC2s) into alterations in membrane excitability and transcriptional programs, rather than relying exclusively on indirect signaling through epithelial or glial intermediates. A well-characterized example involves IL-13/IL-13Rα1 signaling, which enhances neuronal expression of NMU and β-CGRP. ILC2-derived IL-13 stimulates enteric sensory neurons to increase β-CGRP production, which subsequently suppresses ILC2 activity, thereby establishing a reciprocal neuroimmune feedback circuit. Thus, the ENS serves not only as an autonomous regulator of gastrointestinal function but also as an integrative interface at which mechanical, chemical, microbial, and immunological signals converge (Sun et al., 2025).

The functional importance of this immune-sensing capacity is particularly evident during helminth infection. Neuronal deletion of *Il13ra1* impairs host defense against *Heligmosomoides polygyrus* and attenuates immune responses within the muscularis, whereas combined administration of NMU and β-CGRP restores parasite clearance, supporting a causal role for this neuronal pathway in protective immunity (Barilla et al., 2025). In parallel, TLRs and pro-inflammatory cytokine receptors enable enteric sensory neurons to detect microbial products and tissue-damage signals (Benarroch, 2019). Through local enteric reflex circuits and communication with extrinsic neural pathways, these signals can influence motility, secretion, and inflammatory responses.

These findings redefine IPANs as neuroimmune sentinels rather than solely mechanosensory components of enteric reflexes. Dysregulation of IPAN-immune communication may therefore contribute to altered sensory processing, motility, and inflammation in gastrointestinal disorders, including IBD and IBS.

## Altered IPAN signaling may emerge as a convergent feature of gastrointestinal dysfunction

### Disordered motility

Direct causal evidence establishing IPAN dysfunction as a primary cause of gastrointestinal dysmotility remains limited. To date, no peer-reviewed study has demonstrated the complete causal sequence whereby selective impairment of IPAN function produces a defined dysmotility phenotype that is subsequently reversed by restoration of IPAN activity, and comparable evidence is lacking in humans. The strongest functional evidence comes from the identification of *Cdh6* and *Cdh8* as selective markers of Dogiel type II/AH-type sensory neurons in the mouse colon. This molecular definition enabled cell type-specific manipulation, showing that optogenetic activation of *Cdh6*^+^ IPANs evokes retrograde colonic motor complexes (CMCs), whereas pharmacological inhibition of their hyperpolarization-activated current (*I*_h_) disrupts spontaneous CMC generation (Furness et al., 2004). These findings establish that a defined IPAN population is both sufficient and necessary for a specific motor pattern under physiological conditions, but do not demonstrate that pathological IPAN dysfunction causes clinically relevant dysmotility.

Most additional evidence is therefore convergent rather than causal. Foundational studies established IPANs as first-order sensory neurons within enteric reflex circuits and demonstrated persistent changes in their excitability following repetitive stimulation or exposure to inflammatory mediators (Gomez-Frittelli et al., 2025). This capacity for neuronal “sensitization” has been incorporated into mechanistic models of IBS, in which altered sensory processing may contribute to exaggerated enteric reflexes, enhanced recruitment of extrinsic afferents, and visceral hypersensitivity (Smolilo et al., 2020; Vanner et al., 2016). Nevertheless, whether IPAN sensitization is a primary disease driver or a secondary consequence of intestinal pathology remains unresolved (Chang et al., 2018).

Transcriptomic studies strengthen the molecular classification of IPANs but do not establish disease causality. Single-nucleus RNA sequencing of mouse and human enteric neurons identifies IPANs as a distinct transcriptional population among myenteric neuronal subtypes, with characteristic gene-expression signatures (May-Zhang et al., 2021). Such molecular resolution enabled *Cdh6*-based functional studies (Furness et al., 2004), but a stable transcriptional identity in healthy tissue does not indicate whether IPAN states are altered during disease. Importantly, comparable high-resolution datasets specifically characterizing IPANs in patients with IBS or chronic constipation remain limited (Chang et al., 2018).

Pharmacological studies provide clinically relevant but less cell-specific evidence. 5-hydroxytryptamine (5-HT)_4_ receptor agonists, including prucalopride, mosapride, velusetrag, naronapride, and felcisetrag, accelerate gastrointestinal transit (Chedid et al., 2021; Konen et al., 2021; Goldberg et al., 2010), whereas 5-HT_3_ receptor antagonists such as alosetron, ondansetron, and ramosetron can delay transit and increase colonic compliance (Houghton et al., 2000; Fukudo et al., 2016). Activation of 5-HT_1A_ receptors can also inhibit motility (Najjar et al., 2023). However, because these receptors are expressed by multiple neuronal and non-neuronal cell types, these effects cannot be attributed specifically to IPANs. Furthermore, mucosal removal can abolish cyclical 5-HT release without preventing peristalsis, *Tph1* deletion does not necessarily impair transit, and serotonergic antagonists can suppress peristalsis even in 5-HT-depleted tissue (Margolis et al., 2014; Spencer and Keating, 2016; Smith and Gershon, 2015). These findings challenge a strictly mucosal 5-HT-dependent reflex model and support an important role for direct enteric mechanotransduction. Microbiota-dependent regulation of motility through FPR1/ROS signaling and altered myenteric neuronal density in germ-free (GF) mice provides additional evidence for environmental modulation of enteric circuits, although these effects are not IPAN-specific (Chandrasekharan et al., 2025; Chandrasekharan et al., 2019).

Future studies should therefore employ conditional, reversible, and IPAN-restricted manipulations with explicit rescue paradigms, combined with human enteric electrophysiology and disease-specific single-cell or spatial transcriptomics. These approaches will be critical for distinguishing whether maladaptive IPAN sensory coding is a driver of dysmotility, a secondary adaptation, or an epiphenomenon of broader enteric circuit dysfunction.

### Impaired secretory reflexes

To date, no study has established a definitive causal chain linking IPAN dysfunction directly to impaired gastrointestinal secretory reflexes. The most rigorous experimental test, selective genetic, chemogenetic, or optogenetic silencing of IPANs, followed by direct measurement of epithelial secretion and functional rescue, has not yet been reported. Current evidence is therefore convergent rather than strictly causal, arising from morphological, electrophysiological, neurochemical, and pharmacological studies that position IPANs as key first-order sensory neurons within enteric secretomotor reflex circuits.

The anatomical and physiological basis for this role is well established. IPANs initiate intrinsic reflexes regulating both motility and mucosal fluid secretion by transducing mechanical deformation and luminal chemical signals into secretomotor output (Smolilo et al., 2020; Furness et al., 1998). These neurons are present in the myenteric and submucosal plexuses and constitute an important sensory component of local secretory pathways (Benarroch, 2007). IPAN activation is proposed to release excitatory transmitters, including substance P, CGRP, and glutamate, onto cholinergic or VIP-ergic secretomotor neurons, while mucosal stimulation evokes chloride secretion largely through submucosal neural circuits (Weber et al., 2001). Serotonergic signaling provides particularly strong pharmacological evidence for functional segregation within these pathways. Enterochromaffin cell-derived 5-HT activates 5-HT_1P_ receptors on primary afferent neurons to initiate secretory reflexes, whereas 5-HT_3_ receptors on interneurons and secretomotor neurons participate more prominently in peristaltic signaling (Benarroch, 2007; Gershon et al., 1990; Raybould et al., 2004).

Pharmacological studies further support this sensory-relay function. Mechanical stimulation releases nucleotides that activate P2Y1 receptors and induce neurally mediated chloride secretion (Cooke et al., 2004). Intestinal distension recruits stretch-sensitive IPANs together with capsaicin-sensitive extrinsic afferents, which converge on secretomotor circuits involving NK1/NK3, CGRP1, mGluR5, and P2Y receptors (Brookes et al., 2022; Mercado-Perez and Beyder, 2022; Xue et al., 2007). Downstream, VIP and acetylcholine act on epithelial VPAC1 and M3 receptors, respectively, through cAMP- and Ca^2+^-dependent signaling pathways (Karaki and Kuwahara, 2004). However, the transmitter repertoire of IPANs appears regionally and possibly species dependent: tachykinins predominate in the guinea pig distal colon, whereas CGRP has been implicated more strongly in the small intestine, complicating extrapolation to human physiology.

Disease-related evidence remains limited and partly contradictory. Inflammation increases 5-HT availability, enhances AH-type neuronal excitability, and strengthens transmission to secretomotor S neurons, changes predicted to increase neurogenic secretion. Conversely, chronic 5-HT exposure may desensitize receptors on AH-neuronal processes, rendering the net secretory effect uncertain (Vanner et al., 2016). Enteric neuropathies are likewise associated with combined motility and secretory abnormalities, but available studies generally implicate the ENS broadly rather than identifying IPANs as the specific causal locus (Jamka and Gulbransen, 2025). Similarly, IBS mucosal supernatants activate enteric neurons without establishing IPAN-specific causality.

An additional gap is the lack of transcriptomic-functional integration. Although single-cell profiling has refined enteric neuronal classification, no study has directly linked an IPAN-specific molecular signature to an abnormal secretory phenotype. Future work should therefore combine cell-type-specific manipulation of defined IPAN populations with Ussing chamber or *in vivo* secretory measurements, distinguish IPAN defects from downstream secretomotor and epithelial abnormalities, compare acute versus chronic inflammatory states, and validate mechanistic findings in human submucosal preparations. Such approaches will be essential to establish whether IPAN dysfunction is a primary driver of dysregulated gastrointestinal secretion.

## Disrupted neuro-immune communication

To date, no study has formally demonstrated that experimentally induced dysfunction of IPANs directly causes altered neuroimmune communication. The strongest mechanistic evidence comes from Wang et al., who combined genetic and viral approaches with single-nucleus RNA sequencing to identify IL-13R^+^ IPANs as a functional node of gut neuroimmune signaling during helminth infection (Wang et al., 2025). In this circuit, ILC2-derived IL-13 activates IPANs through IL-13R, inducing release of β-CGRP, which in turn suppresses ILC2 responses and limits anti-helminth immunity (Wang et al., 2025). This work establishes a bidirectional neuroimmune circuit in which experimental manipulation alters immune output. However, it does not determine whether intrinsic IPAN pathology, such as degeneration, loss, or genetic dysfunction, initiates maladaptive neuroimmune communication in disease. Thus, an important conceptual gap remains between experimental perturbation of an intact circuit and pathological dysfunction of IPANs.

Transcriptomic studies provide a molecular framework supporting this role. Single-nucleus sequencing has identified a distinct IL-13R^+^ IPAN population that undergoes infection-associated transcriptional remodeling, including increased β-CGRP expression, thereby linking a molecularly defined enteric afferent subtype to immune regulation (Wang et al., 2025). Broader transcriptomic analyses further indicate that IPANs possess molecular signatures distinct from dorsal root ganglion and vagal nodose/jugular sensory neurons, supporting the existence of specialized enteric neuroimmune programs rather than a generic sensory-neuron phenotype (Sun et al., 2025; Nagashima et al., 2019). Nevertheless, these datasets remain largely descriptive and cannot establish whether transcriptional changes actively drive immune dysfunction or instead represent adaptive responses to inflammation.

Receptor-level evidence further supports the capacity of enteric sensory neurons to detect immune signals directly. Primary sensory afferents and related enteric sensory neurons express TLRs and receptors for TNF, IL-1β, IL-6, and IL-17 (Benarroch, 2019). IL-13R is required for neuronal activation by ILC2-derived IL-13 (Wang et al., 2025), whereas RAMP1-CALCRL signaling mediates CGRP-dependent communication between sensory neurons and immune populations, including dendritic cells and ILC2s^64^ (Burgueño et al., 2016). However, receptor expression establishes signaling competence rather than physiological necessity.

Functional studies also reveal substantial context dependence. Antidromic release of substance P, CGRP, and VIP has been associated with vascular permeability, cytokine production, lymphocyte proliferation, and antigen presentation in Peyer’s patches, suggesting immunostimulatory actions (Benarroch, 2019). In contrast, β-CGRP suppresses ILC2 activity in the helminth-response circuit (Wang et al., 2025). These apparently divergent effects may reflect differences in peptide identity, target-cell specificity, inflammatory context, or experimental methodology. Moreover, enteric glia can acquire MHC class I/II expression and antigen-presenting capacity in response to IFN-γ (Brown et al., 2025), emphasizing that IPAN-associated immune effects likely emerge within an integrated neuro-glial-immune network.

Future studies therefore require selective IPAN-restricted loss-of-function models, longitudinal approaches that distinguish neuronal dysfunction as a cause from a consequence of inflammation, and validation in human tissues to determine whether IL-13R^+^ IPAN populations and their signaling architecture are conserved in gastrointestinal disease.

## Microbiota-immune-neuro pathways

No study has yet demonstrated, within a single experimental framework, that gut microbiota dysbiosis causes IPAN “miscommunication” in the strict sense of distorted or corrupted sensory coding. Rather, current evidence primarily establishes a requirement for microbial input in maintaining normal IPAN excitability and circuit function. This distinction is important and warrants caution when attributing causal signal-processing defects to dysbiosis.

Foundational evidence derives from germ-free rodents (McVey Neufeld et al., 2013). Intracellular recordings from jejunal myenteric AH/Dogiel type II neurons in GF mice revealed impaired excitability, characterized by elevated firing thresholds, fewer action potentials at twice-threshold stimulation, reduced input resistance, and a prolonged Ca^2+^-dependent slow afterhyperpolarization. Notably, these abnormalities were normalized following conventionalization of adult GF animals, indicating that microbial colonization can restore key electrophysiological properties of IPANs (McVey Neufeld et al., 2013). Subsequent work extended these observations from individual neurons to functional circuits. Pharmacological excitation of IPANs with the IK-channel blocker TRAM-34 increased mesenteric nerve multiunit discharge by 21% in specific pathogen-free mice and 41% in conventionalized mice, but by only 2% in GF mice. These changes paralleled alterations in sAHP area and the proportion of calbindin-positive myenteric neurons, which was 36% in specific pathogen-free, 24% in GF, and 52% in conventionalized mice (McVey Neufeld et al., 2015). These findings provide the closest evidence for microbiota-dependent “communication failure,” suggesting that signaling from IPANs to extrinsic afferent pathways is markedly attenuated in the absence of microbiota.

Convergent molecular evidence implicates pattern-recognition and microbial metabolite signaling. Enteric neurons and glia constitutively express TLR2 and TLR4, although these receptors are not restricted to IPANs (Jamka and Gulbransen, 2025; Burgueño et al., 2016). TLR2 deficiency produces abnormal neurochemical coding and dysmotility (Obata and Pachnis, 2016; Brun et al., 2013), whereas TLR4 deficiency reduces nitrergic neuronal populations (Jamka and Gulbransen, 2025; Obata and Pachnis, 2016; Caputi et al., 2017), indicating that distinct microbial ligands differentially influence enteric neuronal subtypes rather than exerting a uniform “microbial tone.” Additional receptor-specific mechanisms include microbiota-dependent ENS maturation through serotonergic 5-HT_4_ signaling (Margolis et al., 2021; De Vadder et al., 2018), direct activation of IPANs by commensal-derived N-formyl peptides through FPR1-dependent reactive oxygen species generation (Chandrasekharan et al., 2019), and microbiota-associated regulation of epithelial serotonin production. Spore-forming bacteria and short-chain fatty acids increase *Tph1* expression and improve GF-associated dysmotility (Yano et al., 2015; Yarandi et al., 2020), whereas inhibition of tryptophan hydroxylase with PCPA prevents microbiota-mediated restoration of colonic 5-HT (Yano et al., 2015; Legan et al., 2022).

Important uncertainties nevertheless remain. Colonic 5-HT concentrations in GF animals vary across laboratories and dietary conditions, conventionalization can overshoot rather than simply normalize calbindin expression, and IK-channel pharmacology cannot fully distinguish neuronal excitability from synaptic efficacy. Major gaps include the lack of IPAN-selective genetic manipulation combined with defined microbial communities, reliance on germ-free models rather than clinically relevant dysbiosis, limited sampling beyond murine jejunum, and scarce recordings under physiological luminal conditions. Future studies integrating IPAN-specific conditional genetics, gnotobiotic reconstitution, single-cell profiling, and human enteric neuronal models will be required to determine whether dysbiosis compromises sensory signal fidelity, alters signal gain, or both.

## Visceral hypersensitivity and pain sensation

Whether IPANs directly drive visceral hypersensitivity remains unresolved. To date, no peer-reviewed study has selectively ablated, conditionally disrupted, or optogenetically silenced IPANs while demonstrating that altered IPAN excitability is both necessary and sufficient for enhanced visceral nociception. Current evidence is therefore convergent rather than definitive, arising from electrophysiological, circuit-mapping, transcriptomic, and pharmacological studies. Interpretation is further complicated by the difficulty of experimentally distinguishing intrinsic enteric sensory signaling from extrinsic spinal afferent pathways.

Electrophysiological studies provide the conceptual foundation for IPAN involvement. IPANs, classically identified as Dogiel type II/AH neurons, initiate intrinsic enteric reflexes independently of central input and respond to mucosal chemical signals, including acetic acid and serotonin (5-HT). Repetitive or sustained stimulation can induce prolonged increases in their excitability (Milla, 2001). Because extrinsic primary afferent terminals closely appose enteric ganglia, sensitized IPANs have been proposed to recruit spinal afferent pathways, thereby converting local enteric activity into centrally perceived pain. Infection, tissue injury, mast cell-derived 5-HT, and mucosal inflammation have been suggested as potential triggers (Milla, 2001). However, this remains a mechanistic hypothesis rather than direct evidence of nociceptive transduction by IPANs.

Circuit-level evidence is more compelling but lacks cellular specificity. Optogenetic activation of colonic cholinergic, but not nitrergic, enteric neurons evoke nociceptive-like reflexes through cholinergic signaling to dorsal root ganglion-spinal pathways, and inflammation converts this circuit from innocuous to aversive (Wang et al., 2026). Because IPANs are predominantly cholinergic, these findings are compatible with IPAN sensitization, although the manipulation also targets cholinergic interneurons and motor neurons. Conversely, selective enterochromaffin-cell activation is sufficient to induce acute and persistent hypersensitivity to colorectal distension, which is attenuated by the 5-HT_3_ antagonist alosetron (Ford et al., 2024).

Molecular studies further support sensory sensitization but do not resolve this attribution problem. Reduced colonic catechol-O-methyltransferase activity in postinfectious IBS activates a miR-155/TNF-α pathway that increases excitability in both enteric and dorsal root ganglion neurons (Zhou et al., 2023), while miR-199-dependent regulation of TRPV1 has been associated with visceral pain (Zhou et al., 2023). Numerous receptors and ion channels, including TRPV1, TRPA1, TRPV4, TRPM8, 5-HT3/5-HT4 receptors, GABA-B receptors, CRF-R1, neurokinin receptors, and PAR-2, regulate afferent excitability (Ford et al., 2024; Balemans et al., 2017; Bueno et al., 2007; Brizuela et al., 2026). However, m much of this evidence derives from splanchnic or pelvic extrinsic afferents rather than IPANs (Deiteren et al., 2015). Similarly, 5-HT_7_-dependent Cdk5/mTOR/Cdc42 signaling promotes enteric neurite growth and is associated with increased mucosal innervation, neurotrophin signaling, and GAP43 expression (Lee et al., 2025; Chang et al., 2022), but IPAN specificity has not been established.

Accordingly, the principal knowledge gap is causal and cell-type specificity. Progress will require IPAN-restricted intersectional genetic tools, selective manipulation during behavioral nociception assays, simultaneous intrinsic and extrinsic electrophysiological recordings, and validation in human tissues. Critically, future studies should determine whether IPAN sensitization remains necessary when direct extrinsic mucosal sensory pathways are preserved.

## Summary and conclusion

IPAN dysfunction is increasingly understood not as an isolated defect in gut sensation, but as a broader disruption of intestinal homeostatic surveillance. As first-order sensory elements of enteric reflex circuits, IPANs integrate mechanical, chemical, epithelial, microbial, and immune-derived signals to coordinate motor, secretory, vasodilatory, and defensive responses (Figure 1). Reduced afferent signaling may impair initiation of peristaltic and secretory reflexes, contributing to stasis, constipation, defective mucosal clearance, and prolonged exposure to luminal irritants. Conversely, increased sensory gain can lower reflex thresholds and amplify enteric responses, promoting urgency, diarrhea, and visceral hypersensitivity.

**Figure 1:**
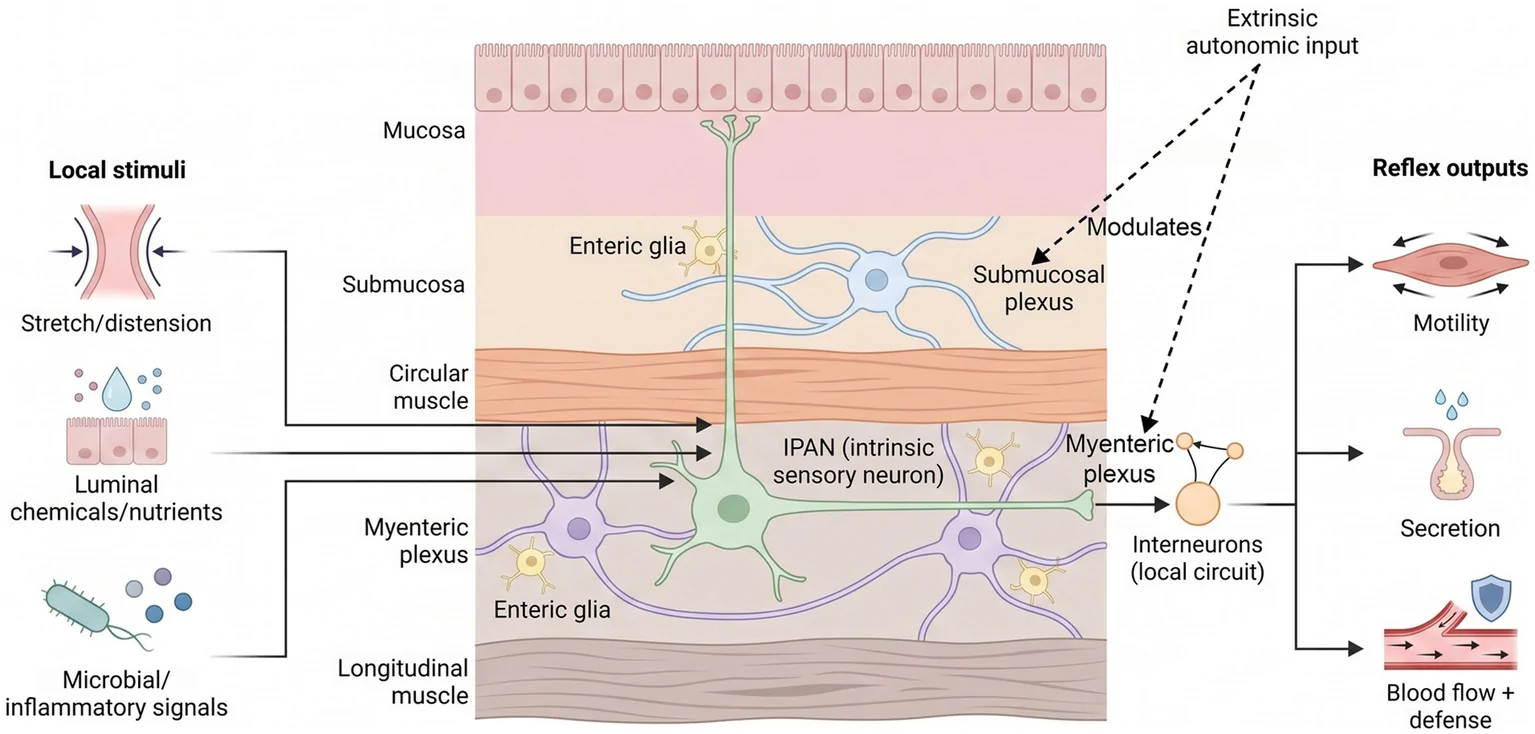
Anatomical structure of the enteric nervous system (ENS) within the gut wall and intrinsic primary afferent neurons (IPANs) as local sensory components that trigger enteric reflexes. The ENS is primarily structured into the myenteric plexus, which governs smooth muscle contraction and motility, and the submucosal plexus, which regulates epithelial secretion, absorption, and local blood flow. IPANs, resembling Dogiel type II neurons, receive inputs from mucosal deformation, luminal nutrients, acidity, microbial metabolites, inflammatory mediators, epithelial signals, and gut wall distension. These signals are transmitted to interneurons, motor neurons, secretomotor/vasomotor neurons, and enteric glia to orchestrate peristalsis, secretion, epithelial transport, vascular responses, barrier support, and defensive reflexes autonomously from direct central nervous system input. Disturbances in IPAN sensory encoding may lead to diminished afterhyperpolarization and increased excitability, thereby amplifying enteric reflexes and extrinsic sensory signaling, which can result in dysmotility, visceral hypersensitivity, abnormal secretion, barrier disruption, inflammation, and altered gut-brain communication.

A central pathogenic mechanism is maladaptive plasticity of neuronal excitability. Suppression of the slow afterhyperpolarization, enhancement of depolarizing currents, and prolonged excitatory synaptic transmission can shift IPANs from adaptive sensory encoders toward persistently hyperexcitable states. This transition may be reinforced by serotonergic signaling, neurotrophins, mast-cell mediators, prostaglandins, cytokines, and reactive enteric glia. Consequently, transient infection or mucosal inflammation may establish a durable form of “neural memory” that sustains dysmotility and hypersensitivity after overt inflammation has resolved. Emerging molecular studies further support this concept by identifying NMU, cadherin-defined Dogiel type II neuronal populations, and IL-13Rα1-expressing IPAN subsets that reciprocally regulate ILC2 responses, thereby positioning IPANs as neuroimmune sentinels rather than purely mechanosensory relays.

Nevertheless, current evidence remains largely convergent rather than definitively causal. Across dysmotility, secretory dysfunction, neuroimmune dysregulation, dysbiosis, and visceral hypersensitivity, no study has yet completed the canonical experimental sequence of selective IPAN disruption, phenotype induction, and functional rescue, particularly in human tissue. Pharmacological approaches often lack anatomical specificity, transcriptomic atlases predominantly define neuronal identity under physiological conditions, and germ-free models establish that microbial input is required for normal IPAN function without proving that dysbiosis directly corrupts afferent coding.

Microbial modulation of enteric excitability is nevertheless well supported, and the ability of rifaximin to reduce visceral hypersensitivity in patients and murine IBS models implicates the microbiota in afferent regulation (Fodor et al., 2019; Lembo et al., 2016; Jin et al., 2018; Xu et al., 2014). Future progress will require IPAN-restricted genetic manipulation with rescue strategies, gnotobiotic reconstitution using defined microbial consortia, and validation in human enteric preparations. Therapeutic recalibration of sensory gain and neuroimmune signaling may ultimately provide a rational strategy for restoring gut homeostasis in chronic inflammatory and functional gastrointestinal disorders.

## Glossary

5-HT: 5-hydroxytryptamine
AHP: Afterhyperpolarization
Calcb: Calcitonin related polypeptide beta
Cdc42: Cell division cycle 42
Cdk5: Cyclin-dependent kinase 5
CGRP: Calcitonin gene-related peptide
CMC: Colonic motor complex
ENS: Enteric nervous system
GABA: Gamma-aminobutyric acid
GAP43: Growth associated protein 43
GF: Germ-free
IBD: Inflammatory bowel disease
IBS: Irritable bowel syndrome
*I_h_*: Inward current
ILC2: Group 2 innate lymphoid cell
IPAN: Intrinsic primary afferent neuron
mTOR: Mechanistic target of rapamycin
NGF: Nerve growth factor
NGFR: Nerve growth factor receptor
NMU: Neuromedin U
TLR: Toll-like receptor
VIP: Vasoactive intestinal peptide
